# Osteopontin as a Biomarker for Coronary Artery Disease

**DOI:** 10.3390/cells14020106

**Published:** 2025-01-13

**Authors:** Georgia R. Layton, Ibrahim Antoun, Alice Copperwheat, Zaidhan Latif Khan, Sanjay S. Bhandari, Riyaz Somani, André Ng, Mustafa Zakkar

**Affiliations:** 1 Department of Cardiovascular Sciences, University of Leicester, Leicester LE1 7RH, UK; 2 Department of Cardiac Surgery, University Hospitals of Leicester NHS Trust, Leicester LE1 5WW, UK; 3 Leicester British Heart Foundation Centre of Research Excellence, Glenfield Hospital, Groby Road, Leicester LE3 9QP, UK; 4 Department of Cardiology, University Hospitals of Leicester NHS Trust, Leicester LE3 9QP, UK; 5 University of Lancaster, Bailrigg, Lancaster LA1 4YW, UK; 6 National Institute of Health Research, Leicester LE5 4PW, UK

**Keywords:** coronary artery disease, osteopontin, acute coronary syndrome, atherosclerosis, calcification, coronary artery bypass grafting

## Abstract

Osteopontin (OPN) is a sialylated phosphoprotein highly expressed in atherosclerosis and upregulated in settings of both acute and chronic inflammation. It is hypothesised that plasma levels of OPN may correlate with the presence of coronary artery disease, “CAD”. This offers potential as a point-of-care testing biomarker for early diagnosis, disease monitoring, and prognosis. This review evaluates the current literature on the association between plasma OPN levels and coronary artery disease and what is currently known to support its potential as a biomarker for future practice. Electronic searches of MEDLINE and EMBASE databases were undertaken from inception until July 2024. Thirty-three studies met the inclusion criteria. All studies were observational, with gross heterogeneity in methods used to analyse the association of plasma OPN with clinical characteristics. They included case series, case–control, cross-sectional, and cohort study designs. OPN has been linked to higher cardiovascular risk and unfavourable cardiovascular outcomes. However, the evidence regarding the direct assessment of CAD severity using tools like the SYNTAX or TIMI scores, which focus on anatomical complexity and risk factors, is less definitive. This suggests that OPN may be a more precise reflection of the inflammatory processes and atherosclerotic activity contributing to unfavourable outcomes rather than a direct indicator of the anatomical severity of CAD itself. Consequently, OPN is increasingly perceived as a marker of a poor prognosis rather than a tool for assessing the severity of coronary artery lesions.

## 1. Introduction

Osteopontin (OPN) is a ubiquitous and multi-functional sialylated phosphoprotein that plays a crucial role in bone remodelling, immune response modulation, tissue repair, and cell survival. Due to its regulatory effects on cell adhesion, migration, and signalling, OPN regulation has already been linked to promoting various inflammatory conditions [1], cancer metastasis [2], and cardiovascular diseases [3]. Whilst reported roles for OPN are wide-reaching across organ systems and are both homeostatic and pathological, its hypothesised or proven roles are linked by the common themes of enhanced expression in response to biological stress or inflammation, as well as its stimulation of both cell motility and cell survival pathways [4]. It is chemotactic for many cell types, notably those essential to cell-mediated immunity such as macrophages, and it is hypothesised to be a provider of anti-apoptotic signals that help cells survive what would otherwise be non-survivable physiological insults; apoptosis events have long been implicated within the progression of CAD [5].

Current CAD prognostic and diagnostic tools, such as coronary imaging and clinical scoring systems, are resource-intensive and may not directly reflect underlying pathophysiological processes like inflammation or atherosclerotic activity.

**Figure 1:**
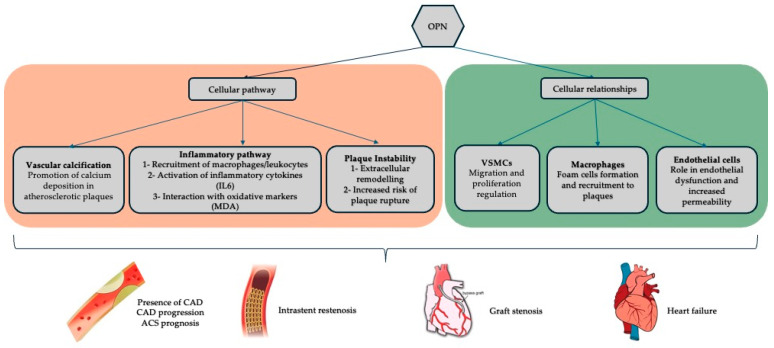
Summary of the currently established relationships of OPN within coronary artery disease physiology and its common treatment modalities (percutaneous stenting and coronary artery bypass grafting). [IL6, interleukin-6; MDA, malondialdehyde; OPN, osteopontin; VSMCs, vascular smooth muscle cells].

In coronary circulation, OPN is produced by vascular smooth muscle cells (VSMCs), such as endothelial cells (ECs), macrophages, and cardiomyocytes. It is considered a key regulator of coronary artery disease (CAD) through its propagation of vascular inflammation through the recruitment and adhesion of macrophages and leucocytes, vessel remodelling through proliferation and migration of VSMC, and regulation of vascular calcification (Figure 1) [6,7]. Furthermore, it can influence plaque vulnerability and risk of rupture, a key feature of acute coronary syndrome (ACS) [8]. OPN is increasingly recognised for its involvement in vascular inflammation, endothelial dysfunction, and plaque instability—key contributors to CAD progression [9]. Plasma OPN levels have been observed to correlate with CAD presence and adverse cardiovascular outcomes in several studies. However, heterogeneity in methodologies, patient populations, and endpoints has hindered the integration of OPN as a reliable biomarker in clinical practice. Additionally, there is some evidence of its role in the development of microcalcification in vein grafts (which can contribute to vein graft failure) used in patients undergoing CABG [10]. Still, its role as a biomarker in patients post-CABG and vein graft disease monitoring is not addressed or understood. This review hypothesises that plasma OPN levels correlate with the presence and progression of CAD and may serve as a biomarker for disease prognosis and risk stratification. By systematically evaluating the available literature, this review aims to determine whether the current evidence supports OPN’s clinical utility as a biomarker for CAD and related conditions, including post-CABG complications.

## 2. Materials and Methods

This systematic review followed guidance from the Preferred Reporting Items for Systematic Reviews and Meta-Analyses (PRISMA) statement standard [11]. A study protocol conforming to the PRISMA protocol was registered at the International Prospective Register of Systematic Reviews (PROSPERO ID CRD42024571553) [12] with the PRISMA checklist reported in our Supplementary Materials.

### 2.1. Study Eligibility

The inclusion criteria were any studies evaluating plasma OPN levels in vivo within human subjects in the context of coronary artery disease or coronary blood flow, which also included an author-defined objective quantification of CAD.

Exclusion criteria included studies of non-human subjects; ex vivo or in vitro study methods; studies evaluating plasma OPN without reference to coronary artery disease or blood flow; studies that did not quantify coronary artery disease within study subjects; studies evaluating OPN in other settings such as in the context of other arteries, i.e., aorta or veins, except for venous conduits used during coronary artery bypass graft (CABG) for the treatment of coronary artery disease; literature reviews, systematic reviews or meta-analyses not reporting original data; conference and meeting abstracts and case reports; and studies not published in English.

### 2.2. Search Strategy

Electronic searches were carried out on EMBASE and OVID MEDLINE using MeSH terms and keywords in subject fields relating to OPN, CAD, myocardial ischaemia, and associated interventions to improve myocardial blood supply. No restrictions based on language were applied to the initial searches. The full texts of any studies with an abstract not published in English were sought before exclusion. Before article exclusion, the reference list of relevant systematic reviews or meta-analyses were reviewed to identify any relevant articles.

From inception until July 2024, electronic searches were conducted using MEDLINE and EMBASE without date or language restriction, with the full search strategy reported in the Supplementary Materials.

The search strategy employed to determine relevant studies utilised combinations of keywords such as “osteopontin”, “coronary artery disease”, “myocardial ischaemia”, and associated interventions to improve myocardial blood supply. A full description of the search strategy is listed in the Supplementary Materials (Table S1). In addition, the reference lists of all retrieved articles were searched for further relevant studies that had not previously been identified.

Only papers with their full text published in English were considered for subsequent analysis. Reviewer G.R.L. performed the database searches. Search results were imported into the Rayyan QCRI web app [13], and duplicates were identified and removed.

To select relevant papers identified by the electronic search, papers were assessed initially by their title and abstracts. Reviewers G.R.L, I.A, and M.Z performed this independently. Conflicts were resolved by consensus discussion between all three reviewers. Studies not excluded after this stage were then examined in full to confirm their meeting inclusion criteria. Authors G.R.L, I.A, and M.Z then validated the final selected papers, and final conflicts were resolved by consensus discussion.

### 2.3. Data Extraction

A standardised form was developed to extract data from the included studies to assess study quality and evidence synthesis. This form was tabulated using Microsoft Excel 2016 (Microsoft, Redmond, WA, USA). Data were extracted from figures, tables, and graphs (using digital image analyser software Webplot Digitizer v5 [14] as necessary) and from the main text. Data parameters (if reported) were collected on this purpose-designed data collection sheet by reviewers A.C and Z.K. For all included studies, these data, where reported, were categorised under the following headings: title, author, year, journal, study design, population demographics and baseline characteristics, validated cardiovascular assessment scores (if used), study methodology (including design, method for quantifying both coronary artery disease, and technique to quantify plasma OPN levels), clinical outcome measures, and plasma OPN levels.

Author G.R.L validated the findings, and discrepancies were resolved by discussion between G.R.L., I.A. and M.Z.

### 2.4. Study Outcomes

The primary outcome measure was plasma OPN level. The quality of the included studies was assessed using the Joanna Briggs Institute critical appraisal tools for observational studies [15]. Following data extraction, reviewer G.R.L. performed quality and risk of bias assessments on all studies. Any discrepancies were resolved by discussion between all authors.

### 2.5. Data Synthesis

A narrative synthesis of all the included studies was performed. Given the anticipated diversity of outcome measures, a limited scope for statistical analysis was expected, so meta-analysis was not undertaken.

## 3. Results

**Figure 2:**
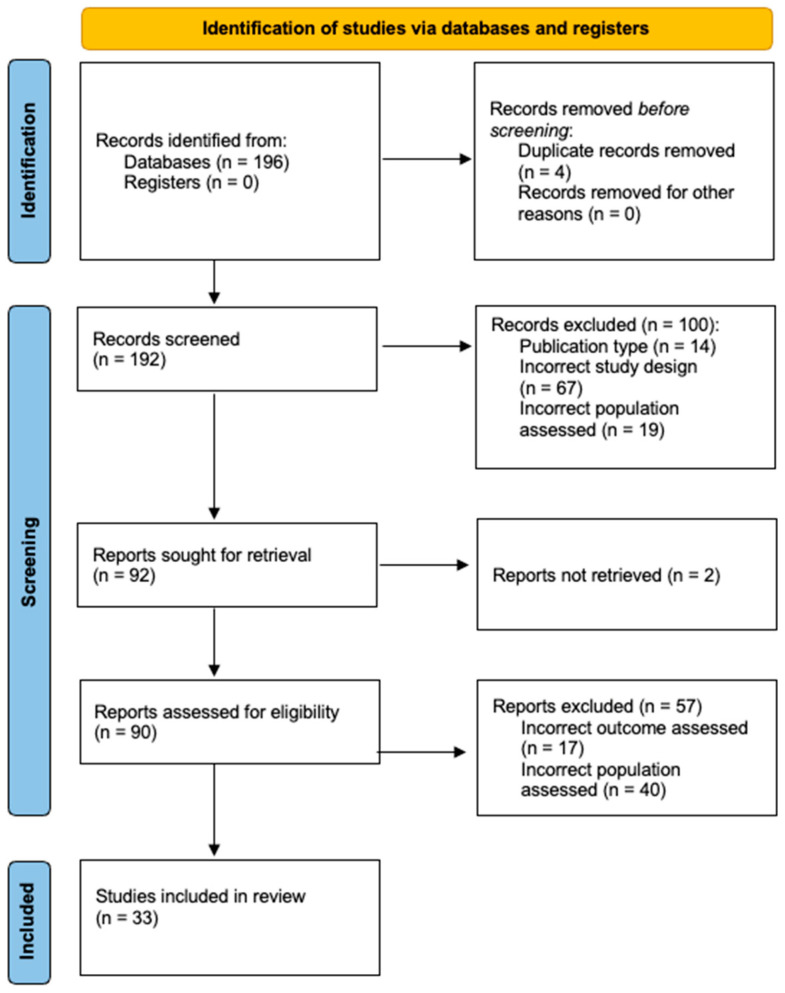
2020 [11] diagram detailing the summary of article assessment and included paper selection.

A total of 196 articles were identified via literature search. In total, 4 were removed as duplicates, and 192 were screened against inclusion and exclusion criteria. Of these, thirty-three were eligible for inclusion in this review (Figure 2). A summary of all included studies is provided in Table 1.

All studies assessed plasma OPN levels in human subjects with quantifiable CAD, including those who had undergone CABG. Included studies were published in the last two decades (2000–2020), with the most historical being published in 2003. All studies were observational, with gross heterogeneity in methods used to analyse the association of plasma OPN with clinical characteristics. Included study methodologies were case series, case–control, cross-sectional, and cohort study designs. These utilised a mix of prospective and retrospective analysis. No randomised or blinded data were reported. Study size ranged from small to large with populations between 50 and 3567 patients.

### 3.1. Bias Assessment

All full texts meeting the inclusion criteria were assessed for bias, utilising a modified version of the Joanna Briggs Institute standardised critical appraisal tools for non-interventional studies [15], as presented in Table 2. Twelve studies were deemed to represent some degree of bias. Given the lack of established, standardised tools for these study types and established interventional data, it is not possible to objectively quantify to what extent these biases may have influenced the outcome data or in which direction. Identifiable bias pertained mostly to a lack of detail in reported methodology or clinical data or unaccounted-for confounding factors, generally due to differing prevalences of non-cardiovascular diseases known to influence OPN levels, such as diabetes.

### 3.2. OPN as a Biomarker for CAD and ACS

A total of 31 studies were included. The pathophysiology of the correlation between CAD and OPN has been explored by Moschetta et al., who assessed circulating OPN and metabolites related to the arginine pathway and oxidative stress using ELISA and mass spectrometry in 25 controls and 33 patients with overt atherosclerosis [28]. Oxidative stress was also suggested as the pathophysiology for the correlation between OPN and CAD. OPN and malondialdehyde (MDA) levels were studied in 71 patients, of which 58 had significant CAD. OPN levels were independently linked to MDA (R^2^ = 0.278, *p* = 0.0004). Furthermore, diabetic patients had higher OPN (73.6 ng/mL versus 56.1 ng/mL, *p* = 0.02) and MDA levels (2.5 μM versus 2.0 μM, *p* = 0.002) [19]. In CAD patients, OPN was positively correlated with 2,3-dinor-8-isoPGF2a (*p* = 0.02), ornithine (*p* = 0.01), ADMA (*p* = 0.001), SDMA (*p* = 0.03), and citrulline (*p* = 0.008), potentially explaining the pathophysiology. A study of 122 patients with spontaneous coronary artery dissection revealed that those with total occlusion (70 patients) had significant OPN compared to those without substantial stenosis (52 patients). Higher OPN levels were also associated with better-developed collateral circulation [43]. The correlation between OPN and angiography-confirmed CAD was significant in 111 patients compared to 97 controls ((72.99 [51.05–103.64]) versus (5.99 [4.26–7.91]), *p* = 0.001) [36]. OPN was also correlated with CAD, as documented by non-invasive imaging. Berezin et al. proposed that elevated OPN in plasma can be considered an independent predictor of coronary calcification, as measured by the Agatston score on computed tomography (CT) in 46 diabetic patients with known CAD (r = 0.418, *p* = 0.009) [26]. A similar study involving 64 patients correlated OPN with high Agatston, with an area under the curve of 0.741 [44]. Furthermore, Aryan et al. assessed OPN levels in 80 intermediate-risk asymptomatic patients. Overall, OPN levels were similar in those with and without coronary calcification. However, in 49 patients not on certain medications, higher OPN levels were found in those with calcification (8.88 ± 2.85 versus 6.79 ± 2.41; *p* = 0.008) [20].

In a prospective study involving 147 patients, OPN was decreased in patients with ACS compared to patients without ACS and showed no correlation with the SYNTAX score (490 pg/mL versus 845 pg/mL, *p* = 0.001) [41]. However, a prospective study involving 30 healthy controls and 30 ACS patients by the HEART and TIMI scores did not yield a significant difference in OPN between cohorts [47]. Cheong et al. studied 666 patients with OPN measurements who were enrolled and followed for 72 months. OPN was correlated positively with ACS-related hospitalisation, where the highest tertile (Tertile 3) of baseline OPN had the highest risk of ACS-related hospitalisation [46]. Carbone et al. categorised 544 patients according to the presence of CAD and cardiovascular risk factor burden. OPN was identified as an independent variable associated with CAD but only in the low-risk-factor group (OR 8.42 [95% CI 8.42–46.83]; *p*-value = 0.015) [33]. Yu et al. compared OPN concentration in 12 ACS patients compared to 16 controls and found that plasma OPN was associated with incident ACS, and the multivariable-adjusted odds ratio (95% confidence interval) was 1.29 (1.06–1.57) per one standard error increase for OPN [30].

OPN was elevated more in ACS than in CCS and was associated with the progression of atherosclerotic disease in a prospective study of 77 patients followed up for 180 days [18]. Another study that assessed CAD progression was the EISNER study, which analysed patients without known atherosclerotic cardiovascular disease. It assessed coronary artery calcification (CAC) and various biomarkers at baseline and after 4 years. Out of 1207 subjects, 621 had a baseline CAC > 0. Among these, 323 progressed, and 121 showed rapid progression. Unadjusted analyses indicated baseline CAD was linked to OPN, TROY, and TNFR1α, but only OPN was independently associated with CAC progression after adjustments [31]. Yu et al. correlated OPN with higher ACS severity and earlier onset time [30]. Furthermore, Abdel-Azeez correlated OPN levels with CAD presence and the number of stenosed coronary arteries by angiogram in 120 patients [34]. This was also supported by Tousoulis et al., who included 409 subjects; 280 with CAD and 129 without CAD patients had higher levels of OPN, which was also associated with three-vessel CAD (0.006), independent of other risk factors [22]. Another study proposed that plasma levels of OPN were independently correlated with the extent of CAD in 301 diabetic patients, of which 226 had angiographically proven CAD [24]. Ohmori et al. further supported these findings by studying OPN levels in 178 coronary angiography patients. Patients with CAD had higher OPN levels (616 ng/mL) compared to those without CAD (443 ng/mL, *p* < 0.001). OPN levels increased with more severe stenosis and were significantly linked to CAD (odds ratio = 1.21). In 86 patients with coronary calcification, OPN levels were also elevated (608 ng/mL versus 490 ng/mL, *p* < 0.01). Furthermore, elevated OPN levels indicated greater CAD severity [16]. In a study of 136 patients, 96 (71%) had CAD, showing higher OPN levels (562 ng/mL) than those without CAD (445 ng/mL, *p* < 0.01). OPN increased with more stenotic vessels, with weak but statistically significant correlations to stenosis (severity > 50%, r = 0.23 and >25% r = 0.24, *p* < 0.01) [25]. However, Coskun et al. did not demonstrate an association between OPN and the extent of CAD, although it was elevated in 65 patients with unstable angina and non-ST elevation ACS [45]. Another study of 52 CAD patients and 30 controls did not correlate OPN levels with CAD [42]. This is particularly important, as it highlights the potential of OPN as a marker of disease presence and as a tool for risk stratification in clinical settings.

Lin et al. evaluated circulating OPN levels and patients with known single-nucleotide polymorphisms of the OPN allele, which have been demonstrated to be associated with higher susceptibility to cardiovascular diseases [27]. In patients with CAD, they found no significant correlation between the presence of specific SNPs and circulating OPN levels. Following survival analysis, they reported an association between higher OPN levels and all-cause mortality over four years (*p* = 0.001). However, there were significant confounding factors amongst their population that was not adequately controlled for; therefore, the correlation between OPN levels and mortality is uncertain.

Moreover, Kwee et al. explored the relationship between OPN and circulating microRNA levels in 186 patients with non-ST-segment ACS. Their study found that elevated OPN levels were associated with adverse clinical outcomes, further supporting its role as a biomarker in ACS [39]. This was also suggested in patients with chronic coronary syndromes in a study where OPN levels were measured in 799 patients with stable angina and CAD. Over a median follow-up of 2.7 years, higher OPN levels (HR 1.88, *p* < 0.001) were significant predictors of adverse cardiovascular outcomes [17]. However, Okyay et al. did not correlate OPN with STEMI and major adverse cardiovascular events (MACEs) [21]. However, OPN levels were higher in 80 STEMI patients compared to 60 healthy controls (23.8 [16.7–41.3] ng/mL versus 18.0 [11.3–31.5] ng/mL, *p* = 0.004). Another study that did not correlate OPN with ischemic extents was conducted by Andrup et al. OPN was measured in serum samples from STEMI patients (n = 239) on Day 1 and Month 4. Ischaemic injury was assessed via various metrics, and all-cause mortality was tracked over a median follow-up of 70 months. OPN declined in STEMI patients during this time without a correlation with the ischaemic injury [40]. According to Yilmaz et al., OPN was associated with intra-stent stenosis after PCI [29]. They studied 91 patients after coronary artery stenting and 60 control patients. After a mean follow-up of 36.7 months, 34.1% of the stented patients developed in-stent restenosis. Their mean plasma OPN level was significantly higher (2721.4 pg/mL, *p* = 0.11) than those without restenosis (1770.4 pg/mL) and controls (1572.4 pg/mL, *p* = 0.002). This was supported by Kato et al., who measured OPN levels in 90 CAD patients undergoing angiography for suspected restenosis, including 52 who had received bare metal stents. OPN levels were also assessed in 60 matched CAD patients without PCI history. Of the PCI patients, 42 had restenosis, showing a higher rate of diabetes and significantly elevated OPN levels (*p* < 0.01), with 38% exceeding 600 ng/mL. Multivariate analysis was an independent predictor of restenosis, with an odds ratio of 1.7 for each 100 ng/mL increase in OPN (*p* < 0.01) [48].

Furthermore, according to Akan et al., OPN was not correlated with troponin levels or TIMI scores [47]. However, another study correlated OPN with poor outcomes. Georgiadou et al. prospectively assessed OPN 101 patients with stable CAD. The primary study endpoint was the composite of cardiovascular death, nonfatal myocardial infarction, need for revascularisation, and hospitalisation for cardiovascular reasons at three years. In a multivariable logistic regression, OPN was independently associated with the primary endpoint (hazard ratio = 2.88, 95% CI: 1.09–7.58, *p* = 0.032) [23].

### 3.3. OPN Role in CABG

Four studies were included. Sbarouni et al. published two case series, likely representing a shared patient population with duplicate data across both papers [35,37]. Their largest series identified 131 patients undergoing CABG [35], with 22.3% (n = 29) having ACS pre-operatively and 4.7% (n = 6) being insulin-dependent diabetics. There was no statistical association between plasma OPN level and patient age, sex, or cardiovascular risk factors such as smoking. OPN levels were higher in those with triple vessel disease (82.3 ng/mL versus 65.3 ng/mL, *p* = 0.23), those with insulin-dependent diabetes (170 ng/mL versus 77.3 ng/mL, *p* = 0.05), or patients with previous acute myocardial infarction (131.5 ng/mL versus 73.3 ng/mL, *p* = 0.007). However, pre-operative OPN levels did not differ between those who went on to have a MACE during follow-up or the hazard of future MACE [hazard ratio (95% confidence interval (CI)): 1.48 (0.43–4.99), *p* = 0.527].

Another study by Cheong et al. [46] presented robust data from the Taiwanese prospective Biosignature registry. This database enrolled patients with a history of acute MI or significant CAD who had received either successful PCI or bypass surgery. Their study, adjusted for confounding factors between groups, identified an association between an elevated OPN level and a higher risk of acute MI-related hospitalisation, especially with an OPN level of more than 4.8 ng/mL. However, the data presented are stratified only by OPN level and do not allow for any sub-group analysis of patients only undergoing CABG. Therefore, the data better represent patients undergoing revascularisation by any method, not surgical revascularisation explicitly. Thus, assumptions regarding the impact of CABG on the OPN level cannot be drawn.

Abdalrhim et al. [38] report that a history of previous CABG is associated with higher OPN levels in patients presenting with stable CAD [regression coefficient (95% confidence interval (CI)): 0.096 (0.061–0.130), *p* < 0.001]. Patients were initially recruited into the PEACE trial to evaluate ACEi versus placebo in patients at least three months following CABG. Within their analysis, no data are provided to describe this cohort of patients or how their confounding attributes, such as stroke, diabetes, and hypertension, compared to those who underwent alternative treatment such as PCI. Furthermore, these patients were presenting with CAD in the context of previous revascularisation, suggesting that the OPN level could be associated with either native new coronary lesions or with stenosis of prior grafts.

### 3.4. OPN as a Biomarker for Heart Failure

Only one study correlated OPN with heart failure. Abdalrhim et al. prospectively examined 3567 patients with stable CAD. The authors demonstrated that OPN was significantly associated with incident hospitalisation for heart failure: HR (95% CI) = 2.04 (1.44, 2.89); *p* < 0.001 [38].

## 4. Discussion

This systematic review’s findings highlight OPN’s promising role as a biomarker for CAD, ACS, and heart failure. The novelty of this review lies in its focus on bridging the gap between biomarker discovery and clinical utility, specifically in the context of OPN and CAD. Unlike prior works that broadly address OPN’s role in cardiovascular diseases, this review systematically evaluates its potential as a biomarker for prognosis and risk stratification while acknowledging its limitations as a diagnostic marker. Moreover, this review emphasises the need for standardisation in measurement techniques and highlights opportunities for combining OPN with imaging modalities, which could provide a more comprehensive assessment of disease activity and progression.

Most of the studies reviewed suggest a significant correlation between OPN levels and CAD presence, extent, and severity [16,22,34]. Studies within this review also consistently indicate that elevated OPN levels correlate with oxidative stress markers, such as MDA and other metabolites within the arginine pathway, which may contribute to the pathophysiology of CAD [28]. OPN levels positively correlate with the Agatston score, suggesting a role for OPN as a marker of coronary calcification and as a potential independent predictor of CAD progression and extent. Moreover, other studies support this association and suggest that elevated OPN may be an independent predictor for coronary calcification, highlighting its utility in stratifying risk within high-risk populations [26].

When evaluating any association between plasma OPN and CAD severity, findings support OPN’s association with more extensive disease, although not necessarily in a directly proportional relationship [16,45]. Multiple studies noted higher OPN levels with greater stenotic burden, establishing an association with the presence and severity of CAD. However, others indicate that OPNs’ correlation with disease extent may vary due to patient heterogeneity, CAD severity, or comorbidities. This suggests a need for further standardisation in research methodologies and stratification of patient populations to navigate OPN’s role fully. Any proportional relationship between OPN and CAD is likely to be heavily confounded by other conditions associated with vascular inflammation and could not be truly identified without prospective, randomised evaluation.

Various studies have assessed the role of OPN in ACS, indicating that elevated levels of OPN may reflect ongoing inflammation and myocardial stress associated with ACS [16,17,19]. For instance, one study identified that higher OPN levels in ACS patients correlate with an increased risk of hospitalisations related to ACS [46], suggesting that baseline OPN levels might have predictive value for future events. Where prospective work has been conducted to evaluate this, it has been shown that OPN levels may demonstrate patterns of expression throughout an ACS event, especially when evaluated in association with other markers of inflammation, such as CRP [30].

However, others showed no significant association between OPN levels and well-established and standardised measures correlating with CAD [47], such as troponin, TIMI, and SYNTAX scores [41]. This highlights the complexity of interpreting OPN levels in the context of ACS, and further research is needed. OPN is a pleiotropic biomarker implicated in various pathological processes, including inflammation, vascular calcification, and immune modulation. While multiple studies have demonstrated a significant association between elevated OPN levels and CAD, it is important to note that OPN lacks disease specificity. Elevated plasma levels of OPN have been observed in a wide range of inflammatory conditions, including autoimmune diseases, malignancies, and chronic kidney disease. This overlap limits its utility as a diagnostic marker for CAD alone. In this review, we emphasise that, while OPN may not be CAD-specific, its value lies in its ability to reflect the inflammatory and atherosclerotic processes that underpin CAD progression and adverse outcomes. Furthermore, combining OPN with other biomarkers or clinical data may enhance its specificity and predictive accuracy for CAD-related events. While OPN is associated with poor cardiovascular outcomes, its role as a marker reflects inflammatory and atherosclerotic activity rather than directly quantifying the anatomical severity of CAD. This distinction is crucial, as poor prognosis may arise from heightened plaque vulnerability and systemic inflammation independent of the extent or complexity of coronary lesions as assessed by tools like SYNTAX or TIMI scores. OPN may, therefore, serve as a complementary biomarker, providing prognostic information beyond anatomical assessments.

The potential of OPN as a predictor for complications after successful CAD treatment is an area of promise for its use. Myocardial blood supply can be compromised after stenting or surgery through the development of both in-stent restenosis and graft disease, respectively. Elevated OPN levels have been demonstrated in patients experiencing in-stent re-stenosis after undergoing PCI. The positive relationship between OPN levels and restenosis indicates its involvement in the inflammatory and fibrotic processes that contribute to restenosis. Nonetheless, additional research is required to determine if OPN could inform post-PCI management or revascularisation strategies.

The potential role of OPN in CABG outcomes remains less established, primarily due to the limited number of studies specifically targeting CABG populations. No consistent relationship was demonstrated among those studies reviewed between preoperative OPN levels and subsequent MACE post-CABG. However, higher OPN levels were observed in patients with multiple vessel disease and insulin-dependent diabetes. The Biosignature registry broadly suggests an association between OPN and adverse outcomes in revascularised patients, although specific CABG analyses are lacking. Consequently, the role of OPNs in predicting CABG-specific outcomes remains inconclusive. Future studies should focus on longitudinal data collection in CABG cohorts to better characterise any predictive role of OPNs in surgical revascularisation, considering the present limited evidence supporting the activation of OPN in vein grafts, which is known to be an inflammatory process that is triggered very early in veins implanted into arterial circulation [10,49]. Furthermore, linking imaging modalities of OPN with circulating levels early can potentially provide a novel way of monitoring the progression of vein graft disease.

Only one study explored the correlation between OPN and heart failure outcomes and found a significant association between elevated OPN levels and heart failure hospitalisation in patients with stable CAD. This finding aligns with OPN’s role in inflammatory and fibrotic processes, which underpin the pathophysiology of heart failure. However, this area’s lack of additional studies limits any definitive statements. This suggests that further research is warranted to assess whether OPN can predict heart failure incidence or progression in CAD patients. The influence of ethnicity and sex on the predictive value of OPN is an area of emerging interest but remains underexplored. Few studies have stratified findings based on these factors, leading to significant gaps in understanding. Available evidence suggests that sex-related differences in cardiovascular disease mechanisms may influence OPN levels and their correlation with CAD. For instance, hormonal variations affecting inflammatory pathways could modulate OPN expression in men versus women, although specific data are lacking. Similarly, genetic variations and lifestyle factors across ethnic groups may affect baseline OPN levels and their association with CAD outcomes. For example, polymorphisms in the OPN gene, which may vary between populations, have been linked to cardiovascular disease susceptibility in a study involving the Mexican population [50]. However, these findings are preliminary and require further validation.

### Limitations

The systematic approach undertaken in this review, which involved robust inclusion and exclusion criteria, yielded only 33 studies for analysis. This limited number of eligible studies underscores the scarcity of high-quality research validating OPN as a definitive biomarker for cardiovascular diseases. The heterogeneity in methodologies, patient populations, and outcomes further complicates the interpretation of OPN’s diagnostic and prognostic value.

This gap in the literature highlights the need for continued investigation using standardised and well-validated methods. Future research should focus on determining whether OPN functions as an early or late marker of cardiovascular pathologies and whether it can reliably stratify risk or guide therapeutic interventions. Prospective, multicentre studies with diverse populations and longitudinal follow-up are essential to confirm OPN’s utility in clinical practice.

While the potential of OPN as a biomarker for CAD is promising, the supporting evidence remains limited. This review highlights that OPN levels lack specificity and are elevated in a broad range of inflammatory conditions, which reduces their reliability as a CAD-specific marker. Current published evidence has methodological inconsistencies and a heterogenous design, preventing meta-analysis. Furthermore, there is an almost complete absence of prospective, interventional, and propensity-matched data. All studies included in this review are observational, with significant confounding factors between populations, such as differing levels of diabetes and heart failure and fluctuating severity of ACS in the populations assessed.

Authors have used differing assays to measure OPN, which, as seen with the widespread use of troponin monitoring, can introduce variation in reporting, unreliable clinical interpretation, and misclassification without standardisation of measurement thresholds. This inconsistency can compromise clinical decision making, as differing assay results might lead to different diagnoses, prognoses, or treatment plans for the same patient. Moreover, standardised assays are essential for validating biomarkers in large-scale studies and for regulatory approval, ensuring that biomarker-based tests are reliable and reproducible. There is the potential degradation of proteins. Prospectively collected samples are sometimes analysed retrospectively, which may lead to denaturation over time.

As a biomarker, OPN shows differing concentration ranges across conditions with significant overlap, limiting its diagnostic utility in patients who typically exhibit multiple synchronous cardiovascular comorbidities [51,52,53]. Given the broad range of conditions that affect OPN and its lack of specificity, it is unlikely to be helpful as a diagnostic marker. Additionally, small fluctuations in OPN levels could introduce complexity and errors into clinical diagnoses, emphasising the need for standardised assays and cut-off values to ensure consistency in clinical practice. OPN might be better suited as a biomarker for monitoring disease status or prognosis or as a predictor of outcomes, such as evaluating the effectiveness of interventions or medical therapies. This application could support the titration of medications to patient-specific optimised levels. Its most valuable role may lie in predictive biomarking, where significant changes in OPN levels could forecast favourable or unfavourable outcomes following specific exposures or interventions.

As a prognostic biomarker, OPN could help predict the likelihood of events such as disease recurrence, stent or graft occlusion after PCI or CABG, or progression of CAD in at-risk groups. However, whilst evidence here suggests higher baseline OPN levels are reflected in enhanced long-term risk of composite cardiovascular outcomes, there is a lack of direct evidence linking plasma OPN levels to specific coronary outcomes. While emerging data suggest a correlation between elevated OPN and proxy measures of coronary ischemia, like the increased severity of CAD [16], there is limited evidence to show that changes in clinical status are reflected by changes in plasma OPN levels [54]—an accurate measure of a biomarker’s utility as a surrogate for disease. Therefore, OPN may be the most useful when combined with other biomarkers, such as troponin or CRP, and clinical data rather than as a standalone marker.

OPN’s specificity for CAD is limited due to its involvement in a broad range of inflammatory and systemic conditions, which may confound its utility as a standalone diagnostic marker. Furthermore, the influence of ethnicity and sex on OPN levels remains underexplored. Preliminary evidence suggests that genetic, hormonal, and environmental factors may affect OPN expression and its relationship with CAD. These findings underscore the importance of stratified research in determining whether demographic variables influence OPN’s predictive value, enabling tailored clinical applications.

The data evaluating OPN levels, specifically in patients undergoing surgical treatment for CAD and CABG, were severely limited. Conduits after CABG, especially vein grafts, are prone to stenosis or occlusion. This process of progressing luminal obstruction, a combination of intimal hyperplasia and accelerated atherosclerosis, is well understood, although its causes are less well known. However, it has been shown that OPN can be over-expressed in these areas and contributes to the development of vein graft disease. We have previously demonstrated in an ex vivo study that 18F-sodium fluoride uptake imaging can be used to localise sites of OPN expression visually [10]. This could allow for direct visualisation of vein graft disease or failure after CABG. Combined with serial measurements of circulating OPN levels, this could provide a role for OPNs, specifically as biomarkers for monitoring patient progress after CABG. Studies included in this review have highlighted the correlation of OPN with oxidative stress markers, such as MDA and arginine pathway metabolites. However, the lack of a complete blood biochemistry analysis in many studies limits the ability to distinguish whether OPN elevations are specific to cardiovascular damage or influenced by comorbid conditions, such as diabetes, renal dysfunction, or systemic inflammatory diseases. This underscores the need for future research to integrate OPN measurement with comprehensive blood biochemistry, including lipid profiles, inflammatory markers, and oxidative stress parameters. Such an approach would allow for the identification of metabolic signatures specific to cardiovascular pathologies while accounting for confounding factors from other comorbidities. Addressing this challenge is crucial to establishing OPN as a particular and reliable biomarker for cardiovascular disease. While OPN has shown promise in reflecting inflammatory and atherosclerotic activity, its utility as a standalone marker is limited by methodological heterogeneity, variations in study cohorts, and the influence of pathological confounders like systemic inflammation, diabetes, and renal dysfunction. These factors underscore the need for rigorous validation of measurement methods and the inclusion of diverse, well-characterised populations in future studies.

Establishing robust diagnostic criteria for OPN requires addressing these limitations through standardised assays, prospective study designs, and controlling for confounders. By doing so, the scientific community can better delineate OPN’s role as part of a multimodal biomarker strategy, enhancing its specificity and clinical utility for cardiovascular disease.

## 5. Conclusions

Overall, plasma OPN has strong potential as a biomarker for CAD, most likely for assessing disease prognosis and predicting adverse outcomes. However, further research and validation are needed before it can be widely adopted in clinical practice. Numerous studies have proposed that OPN has the potential to serve as a biomarker for predicting adverse cardiovascular events. However, its association with CAD severity has shown inconsistent results. OPN has been linked to a higher cardiovascular risk and unfavourable cardiovascular outcomes. Yet the evidence regarding the direct assessment of CAD severity using tools like the SYNTAX or TIMI scores, which focus on anatomical complexity and risk factors, is less definitive. This suggests that OPN may be a more precise reflection of the inflammatory processes and atherosclerotic activity contributing to unfavourable outcomes rather than a direct indicator of the anatomical severity of CAD itself. Consequently, OPN is increasingly perceived as a marker of poor prognosis rather than a tool for assessing the severity of coronary artery lesions.

## 6. Future Perspective

To comprehensively evaluate OPN as a biomarker for CAD, an interventional study is required to assess OPN levels in patients with ratifiable CAD compared to health controls. Stratification of the relationship of OPN levels to atherosclerotic plaque burden, the number of affected vessels, and the degree of stenosis with the support of coronary imaging will also be required. Similarly, further studies are needed to look at the changes in OPN over time, and these can be linked to the imaging of OPN expression in conduits, particularly venous conduits. This will allow for direct visualisation of vein graft disease and failure to OPN expression and has the potential to offer both a biomarker and non-invasive imaging modality for monitoring vein graft progression post-CABG. This can provide a combined biochemical and visual assessment of disease activity. Additionally, exploring OPN as a therapeutic target might uncover new strategies to modulate inflammatory processes and improve clinical outcomes. These advancements could position OPN as a valuable biomarker in CAD management and beyond, but further validation and research are crucial to unlock its full potential.

## Figures and Tables

**Table 1:** A descriptive summary of all included papers.

Authors	Year	Country	Study Setting	Sample Size, Total Males	Population Description	Key Findings
Europe						
Ohmori, R. et al. [16]	2003	Ireland	Prospective, cross-sectional	295	Patients undergoing CAG for suspected CAD	OPN was associated with presence and extent of CAD.
Minoretti, P. et al. [17]	2006	Italy	Prospective, case series	799, 595	CCS patients	OPN levels are an independent prognostic biomarker for patients with CCS.
Mazzone A. et al. [18]	2011	UK	Prospective, cohort	77, 65	CAD patients (ACS and CCS)	Higher OPN levels are correlated with accelerated atherosclerosis in patients with CAD post-PCI. It was more increased in ACS compared in CCS.
Georgiadou, P. et al. [19]	2008	UK	Not mentioned	71, 61	CAD patients	OPN and malondialdehyde were correlated in patients with CAD; this suggests an interaction between OPN and oxidative stress.
Aryan M. et al. [20]	2009	Japan	Prospective, cross-sectional	80, 51	Asymptomatic patients at immediate risk for ACS	Higher OPN levels correlate significantly and independently with CAD.
Okyay K. et al. [21]	2011	Belgium	Prospective, cohort	140, 108	STEMI patients	OPN levels are higher in the first hours of STEMI. There were no differences in MACEs according to OPN.
Tousoulis, D. et al. [22]	2013	Netherlands	Prospective, cross-sectional	409, 338	Patients undergoing CAG for CAD	Both higher OPG and OPN levels associate positively with both the presence and severity of CAD (as measured by number of vessels affected and by the Gensini score).
Georgiadou P. et al. [23]	2010	UK	Prosective, case series	101, 86	Stable CCS documented by angiography	Higher OPN levels were correlated with adverse cardiac outcomes in patients with stable CAD.
Yan, X. et al. [24]	2010	UK	Prospective, cross-sectional	376, 209	Diabetic CAD patients	OPN is associated with CAD in diabetic patients.
Momiyama Y. et al. [25]	2010	Ireland	Prospective, cross-sectional	136, 105	Patients undergoing CAG for CAD	Plasma OPN levels are correlated with the severities of CAD.
Berezin A.E. and Kremzer A.A. [26]	2013	Ireland	Prospective, cohort	126, 74	DM with documented CAD	Higher OPN levels in diabetic patients with asymptomatic CAD are positively associated with atherosclerosis and coronary calcification.
Lin, J. et al. [27]	2019	UK	POS	617	CAD patients	High OPN levels were strong predictors of mortality in CAD.
Moschetta D. et al. [28]	2020	Italy	Prospective, cross-sectional	58, 49	Patients with atheroscelosis	OPN could play a role in the inhibition of endothelial nitrous oxide synthase and in arginase activation in the context of CAD patients.
Yilmaz, K.C. et al. [29]	2018	UK	Prospective, cohort	151, 92	Patients undergoing PCI	Higher OPN levels were correlated not only with CAD but also with in-stent restenosis.
Yu, K. et al. [30]	2019	UK	Prosective, nested case–control	636, 340	ACS patients	Higher OPN levels correlate with higher severity and earlier onset time of ACS
Nandkeolyar, S. et al. [31]	2019	Ireland	Restrospective analysis of randomised control trial data	2414, 1288	Patients without atherosclerosis but have risk factors	OPN levels independently identify progression of atherosclerosis in patients initially free of atherosclerosis, suggesting the utility of OPN in those with subclinical disease.
Brunton-O’Sullivan et al. [32]	2021	UK	Prospective, cohort	140	ACS patients	Increased OPN in ACS patients.
Carbone, F. et al. [33]	2022	Switzerland	Retrospective analysis of prospective, cross-sectional data	544, 318	CAD detected by CT	OPN was associated with CAD.
Americas						
Abdel-Azeez, H.A.-H. and Al-Zaky, M. [34]	2010	US	Prospective, cross-sectional	120, 74	Patients with CP and CAG indication	Higher OPN levels significantly correlated with CAD, lipid profile and CRP, echocardiographic extent of CAD, and atherosclerosis.
Kato, R. et al.	2006	US	Prospective, cross-sectional	150, 129	Patients CAG for stent restenosis	Increased OPN after PCI is associated with intra-stent restnosis.
Sbarouni, E. et al. [35]	2012	US	Prosective, case series	50, 44	CAD undergoing CABG	OPN levels decreased significantly 72 h after CABG and was associated with baseline CRP. It correlated inversely with post-op troponin.
Mohamadpour, A.H. et al. [36]	2015	US	Prospective, cohort	304	CAD patients with >50% stenosis on CAG.	OPN levels are increased in CAD. OPN was not associated with the extent of CAD.
Sbarouni, E. et al. [37]	2016	US	Prosective, case series	131, 117	Patients with stable CAD undergoing elective CABG.	Pre-op levels of OPN in stable CAD patients undergoing elective CABG are higher in those with prior ACS, on insulin, and with higher EuroSCORES.
Abdalrhim, A.D. et al. [38]	2016	US	Prospective, cohort	3567, 2872	Stable CAD patients	OPN levels were associated with a greater incidence of adverse cardiovascular endpoints and hospitalisation for heart failure.
Kwee, L.C. et al. [39]	2019	US	Restrospective analysis of randomised control trial data, case–control	1577, 986	Patients with medically managed ACS	OPN is correlate with increased mortality after ACS.
Andrup, S. et al. [40]	2024	US	Prosective, case series	239, 199	STEMI patients	OPN has a temporal association with multiple parameters of ischaemic injury and can predict long-term adverse cardiac outcomes.
Vega-Rosales, J.A. et al. [41]	2024	Mexico	Prospective, cross-sectional	147, 101	CAD patients undergoing PCI	OPG and OPN levels are lower in ACS patients and there is no correlation with the SYNTAX score.
Asia						
Gocer, K. et al. [42]	2020	Turkey	Prospective, cross-sectional	82, 43	Patients with CAD	OPN was not correlated with the extent or severity of CAD.
Gurses, K.M. et al. [43]	2019	Turkey	Prospective, cross-sectional	122, 78	CCS patients	OPN is associated with coronary collateral circulation.
Uz, O. et al. [44]	2009	Turkey	Prospective, cross-sectional	64	Patients who are having CT assess for CAD	OPN can correlate with coronary artery calcifications.
Coskun, S. et al. [45]	2006	Turkey	Prospective, cohort	108, 77	ACS patients	Plasma OPN level was elevated in ACS but not associated with the extent of CAD.
Cheong, K.-I. et al. [46]	2023	Taiwan	Prospective, cohort	666, 569	CAD patients undergoing PCI or CABG	Higher OPN levels are associated with significant MACE-related admission in CCS patients with a history of successful PCI.
Africa						
Akan A.S. and Ozlu I. [47]	2024	South Africa	Prospective, cross-sectional	90, 57	Patients with chest pain presenting to ED	There is no correlation between OPN and TIMI, heart score, or troponin level.

ED: emergency department. CAD: coronary artery disease. ACS: acute coronary syndrome. OPN: osteopontin. CABG: coronary artery bypass graft. PCI: percutaneous coronary intervention. CCS: chronic coronary syndrome. CP: chest pain. CAG: coronary angiogram.

**Table 2:** A summary of the predicted bias of included studies.

Authors	Modified JBI Critical Appraisal Tools for Systematic Reviews												
	Were There Clear Inclusion and Exclusion Criteria for the Included Populations?	Was There Clear Reporting of Patient Demographics in the Study?	Were Any Comparator Groups Similar and Recruited from the Same Populations?	Were the Exposures Measured Similarly to Assign People to Both Exposed and Unexposed Groups?	Were Exposures Measured in a Valid and Reliable Way?	Were Confounding Factors Identified?	Were Strategies to Deal with Confounding Factors Stated?	Were the Groups/Participants Free of the Outcome at the Start of the Study (or at the Moment of Exposure)?	Were Outcomes Measured in a Valid and Reliable Way?	If Applicable, Was the Follow-Up Time Reported and Sufficient to be Long Enough for Outcomes to Occur?	Was Appropriate Statistical Analysis Used?	Overall Assessment of Bias	Summary of Judgement
Ohmori, R. et al. [16]	Yes	Yes	n/a	n/a	Yes	Yes	No	Yes	Yes	n/a	Yes	Some risk of bias	Significant between-group differences in clinical characteristics
Coskun, S. et al. [45]	Yes	No	Yes	Yes	Yes	Yes	No	Yes	Yes	Yes	Yes	Some risk of bias	Limited reporting of group characteristics forgoing assessment of inter-group differences
Minoretti, P. et al. [17]	Yes	Yes	n/a	n/a	Yes	Yes	Yes	Yes	Yes	Yes	Yes	Low risk of bias	
Kato, R. et al. [48]	Yes	Yes	n/a	n/a	Yes	Yes	Yes	Yes	Yes	Yes	Yes	Low risk of bias	
Georgiadou, P. et al. [19]	Yes	Yes	Yes	Yes	Yes	Yes	No	Yes	Yes	Yes	Yes	Low risk of bias	
Aryan, M. et al. [20]	Yes	Yes	n/a	n/a	Yes	Yes	Yes	Yes	Yes	Yes	Yes	Low risk of bias	
Uz, O. et al. [44]	Yes	Yes	n/a	n/a		Yes	No	No	Yes	Yes	Yes	Low risk of bias	
Georgiadou, P. et al. [23]	Yes	Yes	n/a	n/a	Yes	Yes	Yes	Yes	Yes	Yes	Yes	Low risk of bias	
Abdel-Azeez, H.A.-H. and Al-Zaky, M. [34]	Yes	Yes	n/a	n/a	Yes	Yes	Yes	Yes	Yes	n/a	Yes	Low risk of bias	
Yan, X. et al. [24]	Yes	Yes	n/a	n/a	Yes	Yes	Yes	Yes	Yes	n/a	Yes	Low risk of bias	
Momiyama, Y. et al. [25]	Yes	Yes	n/a	n/a	Uncertain	Yes	No	Yes	Yes	n/a	Yes	Some risk of bias	Significant between-group differences in clinical characteristics
Mazzone, A. et al. [18]	Yes	Yes	No	No	Yes	Yes	No	Yes	Yes	Yes	No	Some risk of bias	Significant between-group differences in clinical characteristics
Okyay, K. et al. [21]	Yes	Yes	No		Yes	Yes	Yes	Yes	Yes	Yes	Yes	Some risk of bias	Unclear inclusion criteria for matched control group with many clinical and laboratory outcomes reported as a single cohort, forgoing complete assessment of any inter-group differences
Sbarouni, E. et al. [35]	Yes	Yes	n/a	n/a	Yes			Yes	Yes	Yes	Yes	Low risk of bias	
Berezin, A.E. and Kremzer, A.A. [26]	Yes	Yes	Yes	Yes	Yes	Yes	Yes	Yes	Yes	Yes	Yes	Low risk of bias	
Tousoulis, D. et al. [22]	Yes	Yes	n/a	n/a	No	Yes	No	No	Yes	n/a	Yes	Some risk of bias	Significant between-group differences in clinical characteristics
Mohamadpour, A.H. et al. [36]	Yes	Yes	No	Yes	Yes	Yes	Yes	Yes	Yes	n/a	Yes	Low risk of bias	
Sbarouni, E. et al. [37]	Yes	Yes	n/a	n/a	Yes	Yes	No	Yes	Yes	Yes	Yes	Low risk of bias	
Abdalrhim, A.D. et al. [38]	Yes	Yes	Yes	Yes	Yes	Yes	Yes	Yes	Yes	Yes	Yes	Low risk of bias	
Yilmaz, K.C. et al. [29]	Yes	Yes	Yes	No	No	Yes	No	No	Yes	Yes	No	Some risk of bias	Significant between-group differences with heterogenous population and in measurement of OPN
Yu, K. et al. [30]	Yes	Yes	Yes	Yes	Yes	Yes	Yes	Yes	Yes	Yes	Yes	Low risk of bias	
Nandkeolyar, S. et al. [31]	Yes	Yes	Yes	Yes	Yes	Yes	Yes	Yes	Yes	Yes	Yes	Low risk of bias	
Kwee, L.C. et al. [39]	Yes	Yes	Yes	Yes	Yes	Yes	Yes	Yes	Yes	Yes	Yes	Low risk of bias	
Gurses, K.M. et al. [43]	Yes	Yes	n/a	n/a	Yes	Yes	Yes	Yes	Yes	n/a	Yes	Low risk of bias	
Lin, J. et al. [27]	Yes	No	Yes	Yes	Yes	Yes	No	Yes	Yes	Yes	Yes	Some risk of bias	Lack of clinical characteristics reported.
Moschetta, D. et al. [28]	No	Yes	n/a	n/a	Uncertain	Yes	Yes	Yes	Yes	n/a	Yes	Some risk of bias	Unclear inclusion criteria for control group with significant between-group differences in clinical characteristics
Gocer, K. et al. [42]	Yes	Yes	n/a	n/a	Yes	Yes	No	Yes	Yes	n/a	Yes	Some risk of bias	Significant between-group differences in clinical characteristics
Brunton-O’Sullivan et al. [32]	Yes	Yes	Yes	Yes	Yes	Yes	Yes	Yes	Yes	Yes	Yes	Low risk of bias	
Carbone, F. et al. [33]	Yes	Yes	No	Yes	Yes	Yes	Yes	No	Yes	n/a	Yes	Low risk of bias	
Cheong, K.-I. et al. [46]	Yes	Yes	Yes	Yes	Yes	Yes	Yes	Yes	Yes	Yes	Yes	Low risk of bias	
Vega-Rosales, J.A. et al. [41]	Yes	Yes	n/a	n/a	No	Yes	No	Yes	Yes	n/a	No	Some risk of bias	Heterogeneous inclusion criteria between groups without clear descriptions of stratification criteria
Akan, A.S. and Ozlu, I. [47]	Yes	Yes	n/a	n/a	Yes	Yes	No	Yes	Yes	n/a	No	Some risk of bias	Significant between-group differences in clinical characteristics
Andrup, S. et al. [40]	Yes	Yes	n/a	n/a	Yes	Yes	Yes	Yes	Yes	Yes	Yes	Low risk of bias	

## Data Availability

The original contributions presented in this study are included in the article/Supplementary Materials. Further inquiries can be directed to the corresponding author(s).

## Supplementary Materials

The following supporting information can be downloaded at: https://www.mdpi.com/article/10.3390/cells14020106/s1, Table S1: Search Strategy.
