# Supplementary material for: Osteopontin as a Biomarker for Coronary Artery Disease
Source: Cells. 2025 Jan 13;14(2):106. doi: 10.3390/cells14020106 (PMC11764379; doi:10.3390/cells14020106)
Supplement: Supplementary file 1 [file cells-14-00106-s001.zip › cells-3354356-supplementary.v6.pdf]

Table S1: Search Strategy

Medline via OVID (21/06/2024)	1 ((heart or cardiac or coronary or cardio*) adj2 (surg* or procedure* or operat* or stent*)).tw.<br>2 ((coronary or vein or venous or angio) adj3 (graft* or bypass or plasty)).tw.<br>3 exp Percutaneous Coronary Intervention/<br>4 exp Coronary Disease<br>5 exp Angioplasty, Balloon, Coronary<br>6 exp Myocardial Infarction<br>7 exp Coronary angiography<br>8 exp Coronary Artery Bypass/<br>9 exp Myocardial Ischemia<br>10 exp Osteopontin/<br>11 1 or 2 or 3 or 4 or 5 or 6 or 7 or 8 or 9<br>12 10 AND 11<br>13 Limit 12 to Humans
EMBASE (21/06/2024)	1 ((heart or cardiac or coronary or cardio*) adj2 (surg* or procedure* or operat* or stent*)).tw<br>2 ((coronary or vein or venous or angio) adj3 (graft* or bypass or plasty)).tw<br>3 Percutaneous Coronary Intervention/<br>4 Coronary artery disease<br>5 Coronary Artery Bypass graft<br>6 exp heart muscle ischemia<br>7 Exp heart infarction<br>8 1 or 2 or 3 or 5 or 6 or 7<br>9 osteopontin.m_titl.<br>11 8 AND 9<br>12 Limit 11 to humans
